# A Mechanical Brain Damage Framework Used to Model Abnormal Brain Tau Protein Accumulations of National Football League Players

**DOI:** 10.1007/s10439-019-02294-1

**Published:** 2019-08-01

**Authors:** M. F. Horstemeyer, P. R. Berthelson, J. Moore, A. K. Persons, A. Dobbins, R. K. Prabhu

**Affiliations:** 1 Department of Mechanical Engineering, Mississippi State University, Starkville, MS 39762 USA; 2 Center for Advanced Vehicular Systems, Mississippi State University, Starkville, MS 39759 USA; 3 School of Engineering, Liberty University, 1971 Liberty Avenue, Lynchburg, VA 24515 USA; 4 Department of Agricultural and Biological Engineering, Mississippi State University, 130 Creelman St., Starkville, MS 39762 USA; 5 Department of Biomedical Engineering, University of Alabama at Birmingham, Birmingham, AL 35294 USA

**Keywords:** Traumatic brain injury, Damage nucleation, Damage growth, Damage coalescence, Internal state variable theory, Fatigue, Overloads, Creep

## Abstract

A mechanics-based brain damage framework is used to model the abnormal accumulation of hyperphosphorylated p-tau associated with chronic traumatic encephalopathy within the brains of deceased National Football League (NFL) players studied at Boston University and to provide a framework for understanding the damage mechanisms. p-tau damage is formulated as the multiplicative decomposition of three independently evolving damage internal state variables (ISVs): nucleation related to number density, growth related to the average area, and coalescence related to the nearest neighbor distance. The ISVs evolve under different rates for three well known mechanical boundary conditions, which in themselves introduce three different rates making a total of nine scenarios, that we postulate are related to brain damage progression: (1) monotonic overloads, (2) cyclic fatigue which corresponds to repetitive impacts, and (3) creep which is correlated to damage accumulation over time. Different NFL player positions are described to capture the different types of damage progression. Skill position players, such as quarterbacks, are expected to exhibit a greater p-tau protein accumulation during low cycle fatigue (higher amplitude impacts with a lesser number), and linemen who exhibit a greater p-tau protein accumulation during high cycle fatigue (lower amplitude impacts with a greater number of impacts). This mechanics-based damage framework presents a foundation for developing a multiscale model for traumatic brain injury that combines mechanics with biology.

## Introduction

Studies from Boston University^3^,^4^,^28^^–^35,38,45 documenting the pathological brain changes of National Football League (NFL) players have led to increased publicity^26^,^47^ and awareness of the complexities of different brain injury types. Both impacts to the head and shock blasts can produce traumatic brain injury (TBI). Moderate and severe TBI are readily diagnosed, but mild TBI (mTBI) may have no objective manifestation in standard clinical MRI scans. Repeated head impacts can in turn lead to chronic traumatic encephalopathy (CTE), which has an etiology that is complex and poorly understood. Essentially, CTE is a neurodegenerative condition characterized by several clinical symptoms that are cognitive and emotional in nature and progressive over time.^29^ Indeed, a puzzling aspect of CTE has been how symptoms may manifest years after military service or an athlete’s career has ended. A definitive diagnosis of CTE depends on analysis of postmortem brain tissue and although sometimes observed in gross brain features, it is observed most clearly in specific microscopic changes: p-tau protein positive astrocytic and neurofibrillary tangles (NFTs), axonal damage, and atypical neurite form (both axons and dendrites). The disease spectrum has been classified into four stages depending on density and extent of damage.^28^

Repeated subconcussive head impacts or a mix of impacts and blast injuries can induce CTE.^29^,^31^ In 2016, the National Institute of Neurological Disorders and Stroke (NINDS) and the National Institute of Biomedical Imaging and Bioengineering (NIBIB) issued a consensus paper that defined the pathology of CTE as an irregular pattern of accumulation of hyperphosphorylated tau (p-tau) protein in neurons and astroglia about the small vessels located within the cortical sulci (McKee *et al.*^31^,^33^,^34^). Supporting criteria for a diagnosis of CTE include the presence of pretangles and NFTs within the superficial layers of the cerebral cortex.^32^ Currently only diagnosable post-mortem CTE occurs when the tau proteins that stabilize the microtubules in the brain become hyperphosphorylated reducing the binding affinity of tau to the microtubules, leading to their destabilization, thus affecting cellular transport through the axon.^42^ However, McKee *et al*.^29^^–^31,33,34 note that the feature that distinguishes CTE from other tauopathies (e.g. Alzheimer’s) is the presence of NFTs in specific regions of the brain, where from our observations large mechanical stress concentrations exist locally, such as in the convolutions of the cortex, periventricular regions, and in subcortical nuclei. Stern *et al.*,^44^ showed that CTE arising from subconcussive repetitive impacts resulted in tau protein entanglements in the brain inducing long term negative effects in athletes, indicating that a mechanical damage threshold exists in which short term or long term healing does not overcome the deleterious effect of the original impact. Furthermore, McKee and Robinson^32^ asserted that mTBIs can induce progressive, long-term debilitating effects, where even just “one TBI event can produce long-term gray and white matter atrophy, precipitate or accelerate age-related neurodegeneration.” Hence, time related degeneration of the brain has been observed. In summary, the aforementioned studies of mTBI from Boston University have connected the microstructure to the accumulation of p-tau in specific areas of the brain.

Three mechanical loading conditions on the brain can be associated with damage: (1) high impact conditions called mechanical overloads; (2) low impact repetitive conditions called mechanical fatigue; and (3) brain age degeneration over time called mechanical creep. Note that mechanical “fatigue” is not medical fatigue; mechanical fatigue includes an external force of a particular amplitude that is cycled at a certain frequency.

*Regarding* applied mechanics, Garrison and Moody^17^ reviewed monotonic damage growth, Suresh^46^ reviewed fatigue damage, and Pihlajavaara^40^ reviewed creep damage. During a monotonic overload, the load amplitude increases for one half cycle with a magnitude that is greater than the fatigue load amplitude and can be directly related to a concussion. In fatigue, the loading cycles (*N*) or reversals (2*N*) occur over time at a particular frequency; hence, the repetitive impact (reversal) frequency is important when considering the onset of CTE as high-amplitude impacts are associated with low-cycle fatigue (LCF) and low-amplitude impacts are associated with high-cycle fatigue (HCF). When a body is subjected to an applied stress over time, “creep” arises from straining and damage. Hence, the time duration of a material under stress is important.

*Five* possible creep stress fields in the brain can be acknowledged: (1) intracranial pressure (ICP), (2) gravity inducing a body force, (3) a local stress field arising from an adjacent damaged local brain region (p-tau) due to local expansions and contractions thus inducing stress gradients on the adjacent material (see Baugh *et al*.^3^ and Harris *et al.*^18^), (4) movement (e.g., walking and running which transfers repeated stresses through the body to the brain), and (5) sleeping horizontally during the night while being upright during the day induces another type of local mechanical boundary condition. Given that damage has started from fatigue and overloads, we assert that creep occurs following structural changes in the brain, which render it vulnerable (see “Discussion” section) as CTE progresses. These three important concepts of mechanics of overload, amplitude and frequency of fatigue reversals, and creep over time are well-known deformation mechanisms in solids, and while brain tissue is more complex than either crystalline solids or polymers, it shares properties with them at particular length scales. For example, microtubules, the site of action of tau-mediated construction and repair, exhibit reduced stiffness under cyclic loading.^43^ Therefore, we shall assume that p-tau accumulation and neurodegeneration can be analyzed in the context of material and mechanical models. Furthermore, failure of a material under overloads, fatigue, or creep occurs because of local stress concentrations…in any material. These stress concentrations occur in local notch root radii of structures that are curved, and in the brain they occur first in the sulci—the negatively curved flexures of the gray matter. In fact, in McKee *et al*.,^28^,^33^ all of the CTE damaged brains analyzed had p-tau at or near the sulci. In the analysis herein of the 77 available pictures from McKee *et al*.,^28^,^33^ we also observed that in the early stages of damage, the greatest p-tau levels were located in the sulcal regions.

McKee *et al.*^28^,^33^ conducted a post-mortem study on 111 NFL players’ brains and observed dark brown/black regions when the NFL player had been concussed or experienced many subconcussive impacts. As McKee and Daneshvar^31^ explain tauopathies alone are not distinguishable based upon the type of loading that occurred to induce brain damage. We recognize that brain damage has multiscale features lower than that of the tau protein entanglements that can include biochemical, chemomechanical, or even electrochemomechanical deleterious effects on the brain. Studies have indicated that injurious mechanical brain impacts lead to injuries on subscales lower than the continuum macroscale, which through a cascade of biochemical reactions has led to different brain injuries.^5^,^6^,^13^,^25^,^39^,^44^ For instance, diffuse axonal injury, a type of TBI, results in axonal wall rupture due to shearing. Furthermore, during such injuries neurons can incur mechanoporation of the phospholipid bilayer membrane.^14^ Neither the lower length scale micromechanical features (changes in extracellular matrix and cytoskeleton) nor any biochemical, chemomechanical, or electrochemomechanical brain damage are within the scope of the present work. The basic cause–effect relationship that we are addressing is the growth and coalescence of damage nuclei *via* a continuum mechanical model.^15^ Hyperphosphorylated tau protein is the observable biomarker that is available from the human neuropathology data, but future animal experiments may well lead to more detailed measures of the intervening mechanisms.

Before proceeding, the term, damage, needs to be described. The term “damage” was first introduced by Kachanov,^24^ who applied an effective stress concept where the damage area fraction operates on the stress to reduce its strength under creep conditions. Based on the Kachanov^24^ notion, Cocks and Ashby^8^ developed an area growth rate equation based upon the tensile hydrostatic stress and effective plastic strain under creep conditions. Bammann *et al.*^2^ then implemented the Cocks and Ashby^8^ damage growth model into a large strain unified-creep-plasticity model and used it to solve many different complex boundary value problems related to monotonic overloads. Later, Horstemeyer *et al.*^1^,^5^,^20^,^22^ developed a damage model in which the area fraction was multiplicatively decomposed into three terms that independently evolved as internal state variables (ISVs) with each of their associated rate equations: (1) crack/void nucleation,^19^ (2) crack/void growth,^21^ and (3) crack/void coalescence.^21^

As such, a mechanical ISV damage model including nucleation, growth, and coalescence rate equations is used to model abnormal p-tau protein accumulation and its damage sequelae associated with CTE. The ISV damage model provides greater understanding of the associated deformation mechanisms that cause brain damage. The following boundary conditions are assumed as follows: fatigue and overloads during football nucleate, grow, and coalesce brain damage as expressed by p-tau pathologies and then continue to increase under mechanical creep conditions over time. Experimental observations found in the Boston University studies^33^,^36^ on the brain damage included 76 professional football players that have been rigorously quantified and used to calibrate the damage nucleation, growth, coalescence, and total damage area fraction in the model. The damage levels for different football player positions are then used to illustrate that the brain damage model could be used to analyze the progression of damage under the three different boundary conditions.

The rest of the paper is organized as follows: “Materials and Methods” section summarizes the methods for analyzing the Boston University experimental data from McKee *et al.*^31^,^33^,^34^ and Mez *et al.*^35^ and also describes the damage model in more detail. “Results” section shows the results of the ISV model correlation with the experimental data from “Materials and Methods” section. Finally, “Discussion” section provides a discussion and summarizes the results.

## Materials and Methods

In this section, we re-analyze the Boston University study of McKee *et al.*^31^,^33^,^34^ and Mez *et al.*^35^ who quantified tauopathy associated with CTE for different former NFL players. We then introduce an ISV model that correlates well known applied mechanics deformation mechanisms to the different stages of CTE damage. Here, we limit our analysis to just the Boston University data recognizing that this might provide limitations on our analysis.

### Boston University Analysis Using Four Stages of Damage

**Figure 1:**
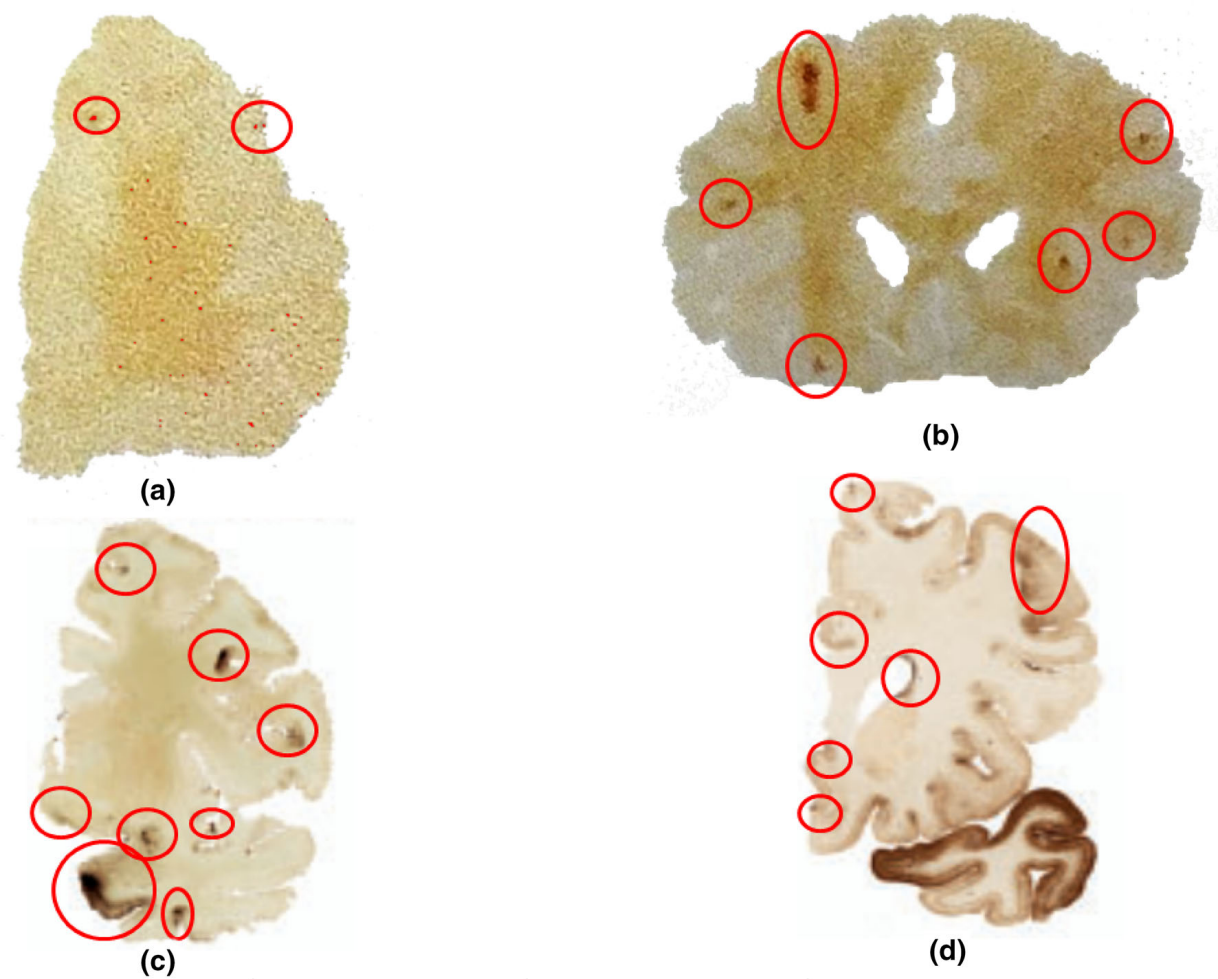
Comparison of tau protein stages found in the brains of deceased athletes analyzed by McKee *et al.*^31^,^33^,^34^ illustrating each stage of damage: (**a**) Stage 1 shows a tau protein area fraction of 0.03% with 2 nucleation sites at an age of 28.3 + 13 years; (**b**) Stage 2 shows a tau protein area fraction of 0.59% with 6 nucleation sites at an age of 44.3 + 16 years; (**c**) Stage 3 shows a tau protein area fraction of 2.87% with 8 nucleation sites at an age of 56.0 + 14 years; and (**d**) Stage 4 shows a tau protein area fraction of 20.85% with 23 nucleation sites (we did not circle them all because there are too many) at an age of 77.4 + 12 years. Note that scales and brain regions differ in the four images.

The Boston University study of McKee *et al.*^31^,^33^,^34^ and Mez *et al.*^35^ analyzed the brains of 202 American football players with 111 of them playing in the NFL. Of the 111 NFL players, 110 exhibited the tauopathy associated with CTE but only 76 pictures were available for our analysis. Histological analyses of the brains revealed dark regions corresponding to p-tau accumulation. Figure 1 illustrates these dark areas and shows the four stages into which McKee *et al.*^31^,^33^,^34^ and Mez *et al.*^35^ categorized the data.

Level 1 (or Stage 1) exhibited the least amount of damage and was associated with an age of 28 years old with data scatter of 13 years; Level 2 incurred more damage and was associated with an age of 44 years old with data scatter of 16 years; Level 3 incurred even more damage and was associated with an age of 56 years old with data scatter of 14 years; finally, Level 4 exhibited the largest area fraction of dark areas associated with p-tau accumulation indicating that these players had incurred the greatest amount of damage. The age associated with Level 4 was 77 years old with data scatter of 12 years. The definition of each level was qualitatively assessed by the Boston University Team.

**Figure 2:**
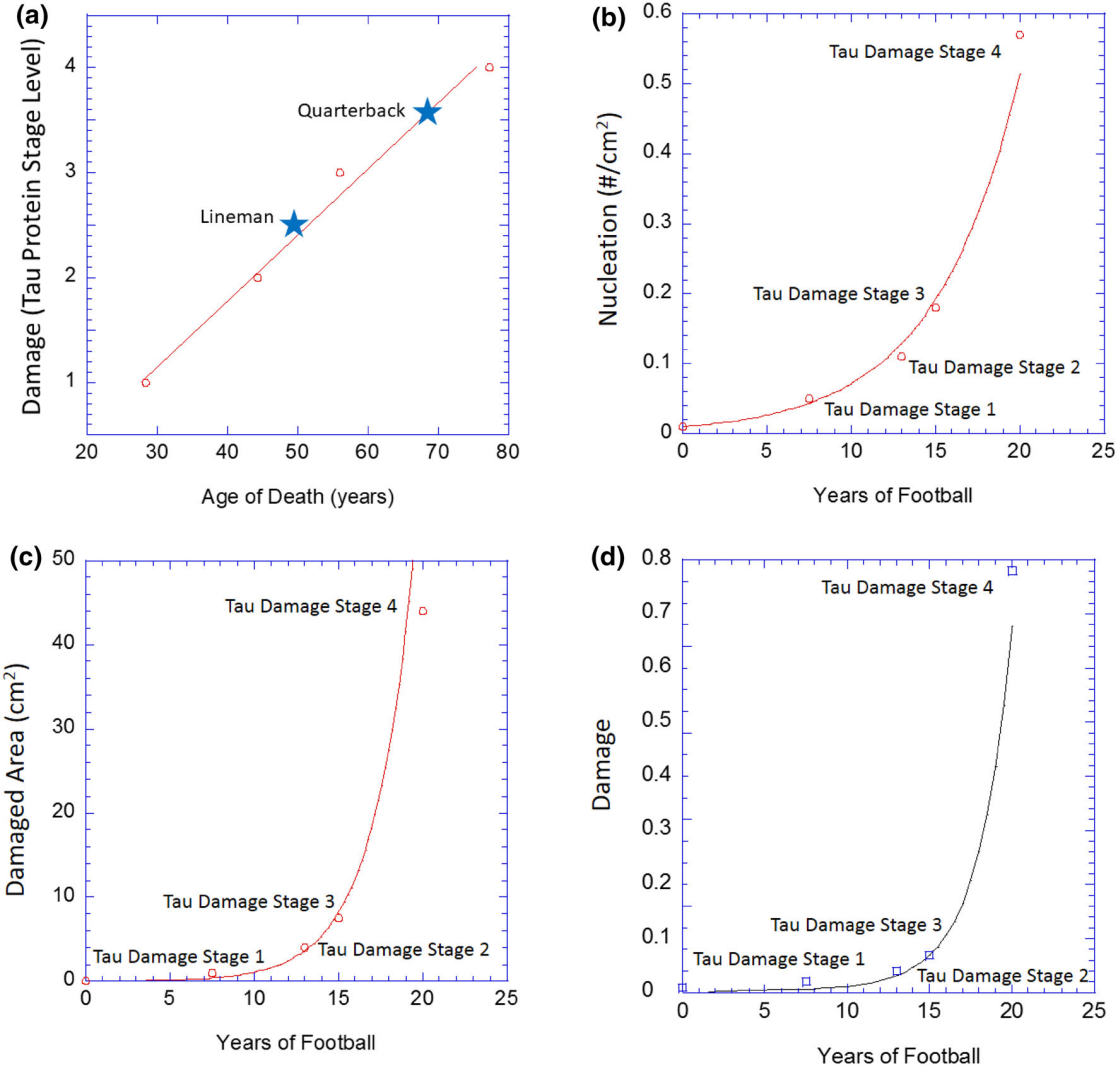
(**a**) The damage level (or stage level) as defined in Mez *et al.*^35^ as a function of the age at death showing almost a linear relationship. Note that as the number of years increases, the damage level increases in almost a linear fashion. We denote two known position players: a lineman (star) and a quarterback (triangle). In parts (**b**)–(**d**) the relevant damage ISV is correlated with CTE stage as defined by McKee *et al.*^31^,^33^,^34^ as a function of the number of years playing football. (**b**) The damage nucleation model. Note that as the number of years of play increases, the damage nucleation level increases exponentially. (**c**) The damage growth model. Damage area also increases in an exponential fashion. (**d**) The damage model showing the area fraction of tau protein damage.

**Figure 3:**
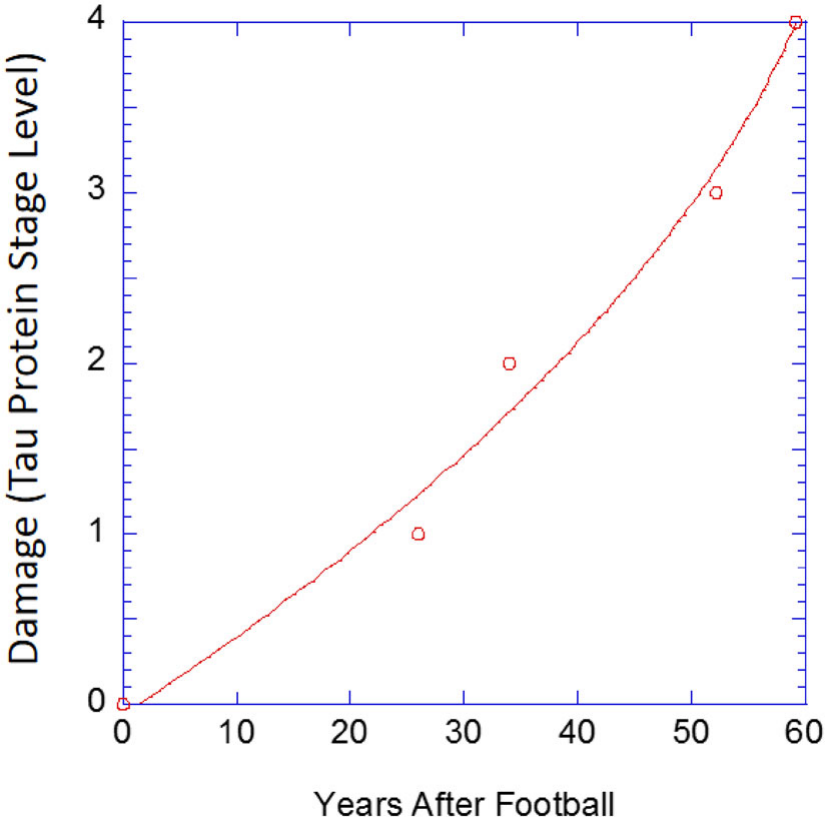
The damage level (or stage level) as defined in McKee *et al*.^31^,^33^,^34^ and Mez *et al*.^35^ during the number of years after a football player had finished playing. Note that as the number of years increases, the damage level increases in a nonlinear tertiary creep fashion.

**Figure 4:**
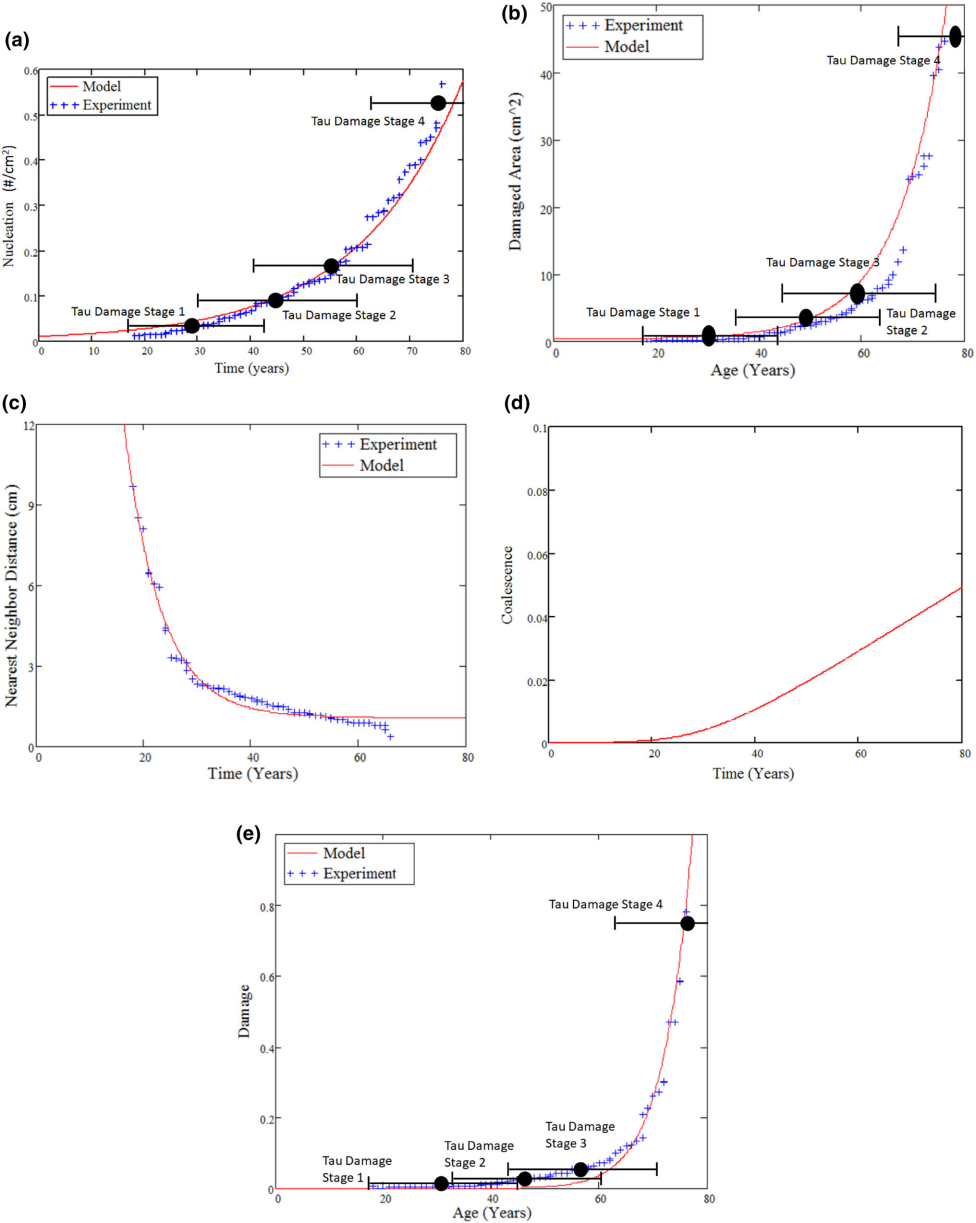
(**a**) The density of damage nucleation sites sorted in ascending order from experimental data. The nucleation model (red line) of Horstemeyer and Gokhale^19^ captures the relationship between the nucleation of tau protein damage sorted in ascending order. (**b**) Damaged tau protein area (mm^2^) signifying the damage growth of tau protein vs. the approximate time at the age of death. The damage growth model (red line) of Horstemeyer *et al.*^20^ captures the relationship between the tau protein damage values sorted in ascending order. (**c**) Damaged tau protein region nearest neighbor distances (cm) signifying the damage interaction of tau sorted in descending order. The nearest neighbor distance between regions of damage relate to the coalescence/interaction model (red line) of Horstemeyer *et al.*,^20^,^22^ which captures the relationship between the tau protein nearest neighbor distance of the tau protein damage when sorted to match the general trends. (**d**) Coalescence (unitless), or interaction, term of the Horstemeyer damage model (Horstemeyer *et al.*^20^) relates inversely to the nearest neighbor distance (*c.f.* Allison *et al.*,^1^) showing the trend as a function of approximate time. (**e**) Total damaged tau protein region (area fraction of tau protein damaged region) vs. the sorted experimental data. The multiplication of the nucleation, growth, and coalescence functions gives rise to the tau protein area fraction curve (red line) (see Horstemeyer *et al.*^20^,^22^). The blue plus signs are individual data points from McKee *et al.*,^33^ and the four stages of tau protein damage are designated that way from McKee *et al*.^31^,^33^,^34^ and Mez *et al*.^35^ (the way we sorted the data removed the tie to the ages given by McKee and Mez).

To quantify the p-tau protein damage throughout the various levels of CTE, the 76 full brain slice images documented in McKee *et al.*^31^,^33^,^34^ were digitized. ImageJ software (Source: NIH, https://imagej.nih.gov/) was then used to create global thresholding restrictions to determine the damage area and the total area of each brain slice image, which were both approximately converted from pixel density to cm^2^. Additionally, the nucleation (#/cm^2^) of each brain slice were calculated using

1 $$ {\text{Nucleation}} = \frac{{\# \;{\text{of}}\;{\text{damaged}}\;{\text{regions}}}}{{A_{\text{Total}} }}, $$

where *A*_Total_ is the total area of each slice. The nearest neighbor distances (*NND*s) between each tau protein damage area were then calculated using ImageJ and the “Nearest Neighbor Distances Calculation with ImageJ” plugin.^23^ Finally, the damage (%) was calculated using

2 $$ {\text{Damage}}\; ( {\text{\% )}} = \frac{{A_{\text{Damage}} }}{{A_{\text{Total}} }}, $$

where *A*_Damage_ is the previously determined damage area and *A*_Total_ is the total area of each brain slice image. As McKee *et al.*^31^,^33^,^34^ did not provide the ages at death for the individual brain slice images, the values for nucleation, damaged area, and damage (%) were arranged in ascending order and assigned approximate ages (years). Similarly, the *NND* data set was arranged in descending order and assigned approximate ages (years). For Figs. 2a–2d and 3, a single data point from each CTE stage was selected to represent these groupings to demonstrate the fitting of the ISV model to the nucleation, damaged area, and damage (%) data sets. The full nucleation, damaged area, *NND*, and damage (%) data sets were then fit to the ISV models in Figs. 4a–4d and 4e, respectively.

Figure 2a illustrates that the tau protein stage levels are linearly related to the approximate age of death indicating a clear correlation of the damage level to the age of the person. Only two data points were of known individuals: a lineman and a quarterback (QB) as shown in Fig. 2a.

As Fig. 1 illustrates pictorially in two dimensions the four different damage level stages, Figs. 2b–2d express the four damage levels with respect to the years of playing football in terms of the three ISVs: damage nucleation (number density of tau protein accumulation sites), damage growth (damaged area associated with abnormal p-tau deposition), and damage (area fraction of tau protein accumulation). Table 1 summarizes the values for the damage model parameters. The damage (area fraction) equals the nucleation multiplied by the growth values^20^,^22^ that were garnered using a best-fit algorithm. Note that in Figs. 2b–2d that the accumulation of p-tau protein damage grows exponentially during years playing the game, indicating that it corresponds to fatigue mechanical loading with many impacts over many cycles (plays). From a mechanical loading perspective, one can think of the years of football with repetitive impacts as fatigue, creep–fatigue, or creep–fatigue with some overloads. As mentioned earlier, fatigue typically has a periodicity associated with it or at least quasi-periodicity. Creep–fatigue occurs when there is a longer time period between a series of fatigue cycles. Regarding football, one could argue that each game induces fatigue reversals but the week between football games induces creep behavior. Creep–fatigue followed by overloads occurs when a random, high magnitude, low frequency impact occurs in addition to the creep–fatigue history.

**Table 1:** Modeling data from damage nucleation, growth, coalescence, and total damage.

Damage model feature	Damage model constants	Years of football (fatigue/overload years) + years after football
Nucleation (*h*) = *f*(*t*)	Coefficient	0.01
	Exponent	5.065
Growth (*v*) = *f*(*t*)	Coefficient	0.016
	Exponent	0.105
Nearest neighbor distance = *f*(*t*)	Coefficient	117
	Exponent	− 0.145
Coalescence (*c*) = *f*(*NND*)	Coefficient	1.15
	Exponent	2.90

Figure 3 shows that the damage growth occurring after the end of the football career is nonlinear. Although the rate of damage growth increases during the years of playing, the damage rate clearly slows down when the impacts cease, which is related to the mechanical loading of creep. One can think of “short term creep” as during the season between games or practices or even during the off-season, but “long term creep” is described herein after as the life of a player post-football. Three phases of creep exist: (1) primary creep, (2) secondary (steady state) creep, and (3) tertiary creep. Based on the data from Boston University, the curvature of tertiary creep matches the trend for the p-tau protein accumulation and damage to the brain. In tertiary creep, material straining grows exponentially. Physically, the material is extending, compressing, or shearing. During a player’s years of football, fatigue, creep–fatigue, or creep–fatigue with overloads has occurred thus initializing the “years after football” with some p-tau protein accumulation damage state. Once the p-tau protein accumulates and damages the brain material, local stress concentrations along with the gravitational body forces will increase the damage over time causing enhanced straining.

### Use of a Continuous Damage Model for Examination of Tau Protein Accumulation and Damage

As Garrison and Moody^17^ reviewed the damage of different solid materials and identified three components of damage that include (1) nucleation, (2) growth, and (3) coalescence of the damaged material regions where a stress field interaction occurs. Horstemeyer *et al.*^20^,^22^ developed the ISV mathematical functions for the separate nucleation, growth, and coalescence terms which multiplicatively give the damage (area or area fraction of the damaged region). As mentioned previously, this damage framework has been used on a variety of materials. Herein, we assume that the association the formal ISV damage model with the p-tau protein accumulation (as a biomarker of brain damage) is appropriate.

Instead of coarse coding into four stages that the Boston University Team employed for the progressive damage states, we reorganize the data as one continuous stream of data by sorting all points by the general trend seen in the four stages. This allows for easier correlations to the ISV damage variables. The ISV nucleation model of Horstemeyer and Gokhale^19^ is modified and simplified for application to model p-tau accumulation and damage in the brain, and the integrated form of the model is given by the following equation:

3 $$ \eta (t) = \eta_{\text{coeff}} [\exp (M\varepsilon (t))], $$

where *C*_coeff_ is the coefficient to the equation, and *M* is a complicated term that includes the microstructure and stress-state dependence. Because of the lack of knowledge of the subscale information associated with the regions of p-tau protein accumulations, the *M* parameter is yet to be related to microstructural features. Furthermore, a hydrostatic tension for the stress-state dependence locally is assumed; hence, even when compression occurs as a boundary condition, locally there is tension because of the Poisson ratio.

The equation used for damage growth is similar to the nucleation equation and is given by the following:

4 $$ v(t) = v_{\text{coeff}} [\exp (Z\varepsilon (t))], $$

where $v_{\text{coeff}}$ is the coefficient to the equation, and *Z* includes the microstructure and stress-state dependence similar to the nucleation equation.

The equations for the *NND* and coalescence are given by the following:

5 $$ NND ( {\text{t)}} = NND_{\text{coeff}} [\exp (Q\varepsilon (t))], $$

6 $$ \dot{c}(t) = C_{\text{coeff}} \left[ {\left( {\frac{4d}{NND(t)}} \right)^{\zeta } } \right];\quad c(t) = \int {\dot{c}(t)} dt, $$

where $NND_{\text{coeff}}$ is the coefficient to the nearest neighbor distance equation, *Q* includes the microstructure and stress-state dependence similar to the nucleation equation, *C*_coeff_ is the coefficient to the coalescence equation, and *d* is the square root of the area damaged by p-tau accumulation.

The multiplication of the ISV nucleation, ISV growth, and ISV coalescence together gives rise to the total damage, which is the area fraction curve as shown in the following equation from Horstemeyer *et al.*^20^,^22^:

7 $$ \phi (t) = \eta (t)v(t)c(t). $$

## Results

We correlate the physics-based ISV model with the sorted data of the Boston University data regarding CTE p-tau pathology and we relate the data and damage mechanisms to football player positions.

### ISV Damage Model Shows Strong Correlation to Tau Protein Damage Progression

Figure 4 shows the correlations of each damage quantity compared to the Boston University tau protein data.^33^ Figure 4a shows the number density [number of regions of p-tau accumulation per unit area (cm^2^)] as a function of approximate time of death for the 76 specimens with pictures examined in the Boston University study.^33^ When *η*_coeff_ equals 0.01, and *M* equals 5.338, a close correlation of the damage ISV nucleation model to the tau protein pathology garnered by the Boston University Team exists (Fig. 4a). Additionally, Fig. 4a also shows where the four different damage levels defined by the Boston University Research Group lie on the plot.

Figure 4b shows the area (cm^2^) damaged by p-tau protein deposition signifying the damage growth of tau protein spots as measured on the brains of the deceased NFL players in the Boston University study^33^ vs. the time at the age of death. The ISV damage growth model of Horstemeyer *et al*.^20^,^22^ correlates well the relationship between the damage incurred by abnormal tau protein accumulation vs. time. When $v_{\text{coeff}}$ equals 0.016, and *Z* equals 0.105, an excellent correlation of the damage growth model to the tau protein pathology garnered by the Boston University Team is found.

Figures 4c and 4d relate to the coalescence of damage that arises when the stress concentrations of nearby damage regions affect their neighbors. As such, the *NND* needs to be quantified. Horstemeyer *et al.*^20^,^22^ and Lawrimore *et al.*^27^ have shown that when the damaged regions are within five diameters (diameter is defined as the square root area of the damaged region) of each other, the damage can accelerate. Figure 4c shows the *NND*s (cm) within the region damaged by p-tau deposition signifying the damage interaction of tau protein measured on the brains of the deceased NFL players analyzed in the Boston University study^33^ vs. the time at the age of death. Figure 4d shows that the ISV coalescence (unitless), or interaction, term of the damage model^20^ relates inversely to the *NND* (*c.f.* Allison *et al.*^1^) showing the trend as a function approximate age of death. When *NND*_coeff_ equals 9.36, and *Q* equals - 0.114, an excellent correlation of the *NND* model to the tau protein pathology noted by the Boston University exists (Fig. 4c). When *C*_coeff_ equals 1.15, *d* equals 0.024, and *ς* equals 2.9, the coalescence evolution shown in Fig. 4d is found.

Figure 4e shows the total area damaged by accumulation of p-tau (area fraction of region damaged by p-tau) as measured on the brains of the deceased NFL players analyzed in the Boston University study^33^ vs. the time at the approximated age of death. The damage model shows a clear correlation with the tau pathology data as illustrated in Fig. 4e. This correlation indicates that the robustness of the multiplicative decomposition in terms of the damage nucleation, growth, and coalescence is strong when used as a damage model for the pathology associated with p-tau deposition.

### Analysis Shows Strong Correlation of Mechanical Loading Conditions to Player Positions

Only a few studies have focused on brain damage related to player positions. For example, Pellman *et al.*^39^ studied NFL players over 6 years (1996–2001) and found that wide receivers (WRs), defensive backs (DBs) and tight ends (TEs) incurred 3.1 concussions per 100 game-positions resulting in the highest number of concussions when compared to all positions. QBs were next, experiencing 1.62 concussions per 100 game-positions. These concussion rates suggest that greater impact forces are experienced by the WR/DB/TE positions as compared to the other positions indicating that either LCF and/or monotonic overload conditions led to the concussions.

Dick *et al.*^12^ conducted a seminal study on the concussion rate per player position. Results of this study showed that 11 concussions occurred per 1000 athletic exposures (aes) (meaning one game or practice, not just one impact) in 16 years (1988–2004) of data from National Collegiate Athletic Association (NCAA) college football. (Note that there is typically more than one impact per game or practice.) Dick *et al.*^12^ found that the greatest amount of concussions occurred at roughly the same rate for the three categories: TE/WR/DBs incurred 28%, while running backs and linebackers (RBs/LBs) incurred 29%, and QBs incurred 28%; however, the linemen incurred only 15% of the total concussions. The conclusion is similar to Pellman *et al.*^39^ in that the skill positions, such as QB garner more concussions (mechanical overloads and/or LCF conditions) than linemen positions.

Funk *et al.*^16^ studied Virginia Tech football players over a 4-year period in which they used accelerometers in the helmets of the players to measure the G-levels of impacts. Results of this study showed that the linemen garnered the greatest number of head impacts but usually at a smaller G-level when compared to the other positions—indicating an HCF regime. Conversely, the other positions (RB/LB, WR/DB/TE) where the impact speed was greater incurred more severe head impacts (peak accelerations > 100 g). Therefore, the conclusions of Funk *et al.*^16^ concur with those of Pellman *et al.*^39^ and Dick *et al.*^12^ that the skill positions exhibited overloads and/or LCF mechanical behavior, while the linemen experience an HCF, low-magnitude impact regime.

The results of Funk *et al.*^16^ were further corroborated by Baugh *et al.*^3^ and Nathanson *et al.*^37^ Baugh *et al.*^3^ studied the incidence of concussions for different player positions in NCAA players and found that symptoms of dizziness, headaches, or “seeing stars” occurred mostly to the linemen indicating that the subconcussive impacts experienced by linemen can cause brain damage reflective of HCF. Furthermore, offensive linemen, in particular, experienced more frequent, low-magnitude head impacts that were not reported as concussions vs. QBs who experienced less frequent, high magnitude head impacts.

**Figure 5:**
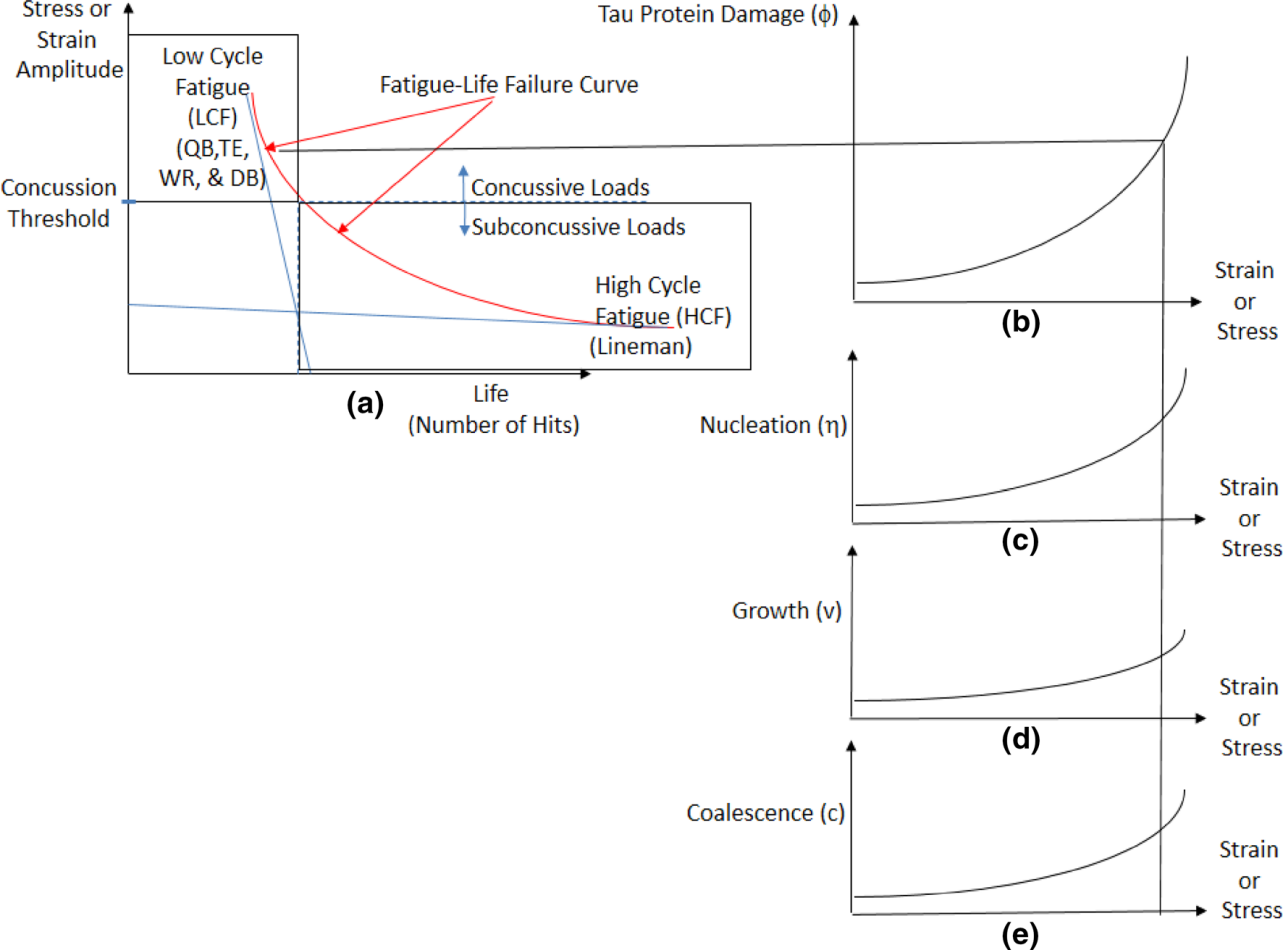
(**a**) An idealized CTE fatigue-life damage curve representing material failure. Notice that as the strain amplitude (or stress amplitude) decreases, the number of impacts increases necessary for material failure in the brain. For low cycle fatigue (LCF), quarterbacks (QBs), the tight ends (TEs), wide receivers (WRs), and defensive backs (DBs) incur a lower number of impacts to failure but experience much greater amplitudes. However, for high cycle fatigue (HCF), the linemen incur a greater number of impacts with lower load amplitudes to realize failure. The transition from LCF to HCF determines the amplitude threshold for concussive and subconcussive impacts. The fatigue failure curve for each level of damage type is associated with a certain damage level with respect to the strain or stress amplitude. Damage (**c**) nucleation; (**d**) growth; and (**e**) coalescence is shown under the applied strain or stress associated with (**b**) the total damage curve and (**a**) the strain life curve.

The Boston University studies^33^,^36^ also confirmed that more linemen garnered brain damage than other player positions. Although the findings of McKee *et al.*^31^,^33^,^34^ and Mez *et al*.^35^ initially appear contradictory to the findings of Pellman *et al.*,^39^ Dick *et al.*,^12^ and Funk *et al.*,^16^ the latter studies focused only on seasonal *in vivo* concussion incidence with no pathological analysis of the brain; whereas, the former studies examined the brain post-mortem to assess the totality of the pathological changes (damage) incurred by the players. Nevertheless, the differences between the skill player positions and linemen resides in the fact that LCF and HCF regimes are being exhibited, respectively, as illustrated in the fatigue-life curve shown in Fig. 5.

## Discussion

While a player at any position can experience an LCF monotonic overload (i.e. concussion), a couple of trends correlating player position to the abnormal accumulation of p-tau can be discerned. Based upon the data of Pellman *et al*.,^39^ Dick *et al*.,^12^ Funk *et al*.,^16^ and Baugh *et al*.,^3^ the QBs and other skill positions can be categorized in mechanical loading terms under fatigue as LCF, while the linemen can be categorized as HCF brain damage. Figure 5 illustrates the strain-life curve of the damage from p-tau accumulation as a fatigue-life failure curve. In this context, mechanical “failure” is defined as any form of CTE.

Although greater magnitude loads could occur in a blast or a car crash, where brain tissue tearing or arterial tearing could arise from very large mechanical loads, the football related damage events are more related to a CTE threshold as denoted by the black line in Fig. 5. Note that a similar amount of p-tau accumulation can arise for a lineman compared to a QB/WR/DB/TE, even though linemen experience much lower amplitude forces due to the lower impact velocities. However, the greater frequency of hits experienced by linemen relative to the skill positions can result in similar damage level on the failure curve as those of the QB/WR/DB/TE positions. In essence, the “failure” curve on the strain-life fatigue curve encompasses the different parameters that have been examined in p-tau pathologies, concussion studies, and subconcussive impact studies. These variables in the fatigue curve include: amplitude of loading associated with the impact velocity, number of impacts, frequency of impacts, and p-tau protein accumulation levels.

Also of note, the LCF regime transitions to the HCF regime at the point where the plastic deformation asymptote intersects the elastic deformation asymptote (which are both designated by the dashed lines). Given this information, the high amplitude impacts experienced at the positions of QB/WR/DB/TE occur within the LCF regime, while repetitive cycles of low amplitude impacts result in HCF failure, like those experienced by linemen.

### Offensive Linemen

**Figure 6:**
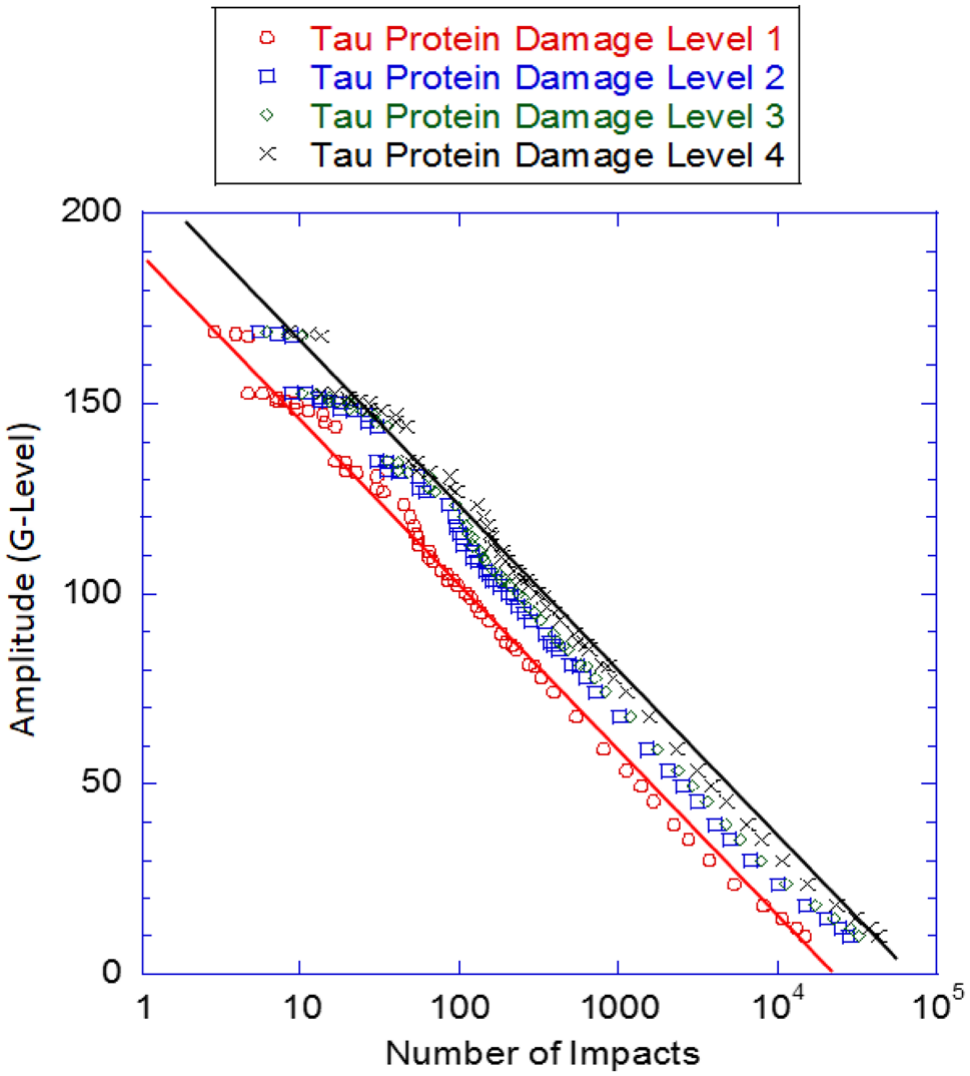
The fatigue-life curve of an offensive lineman showing the different levels of p-tau protein accumulation from the different stages defined by McKee *et al.*^31^,^33^,^34^ and Mez *et al.*^35^ Data for the plots were garnered from Mihalik *et al.*,^36^ Funk *et al.*,^16^ McKee *et al.*,^31^,^33^,^34^ and Mez *et al.*^35^

Figure 5 shows the strain-life curve for an offensive lineman illustrating the damage level associated with p-tau accumulation. From Baugh *et al.*,^3^ offensive linemen, when compared to any other position, incurred the most incidences of symptoms associated with high magnitude impacts or the greatest number of head impacts. Mihalik *et al.*^36^ showed that offensive linemen do experience the greatest number of head impacts over a season; therefore, the symptoms exhibited by the linemen most likely arose from damage incurred in the HCF regime. The p-tau accumulation damage levels from McKee *et al.*^31^,^33^,^34^ and Mez *et al.*^35^ are shown in Fig. 6. Further, the years of football played came from Mez *et al.*,^35^ who divided the damage levels into two stages instead of four stages. An assumption was made that the lowest stage of McKee *et al.*^31^,^33^,^34^ and Mez *et al.*^35^ could be subdivided into Stages 1 and 2 of McKee *et al*.,^31^,^33^,^34^ while the highest stage of Mez *et al.*^35^ highest stage could be subdivided into Stages 3 and 4 from the study by McKee *et al*.^31^,^33^,^34^ It was further assumed that the lowest standard from McKee *et al.*^31^,^33^,^34^ and Mez *et al.*^35^ could be used for Stage 1 (7 years of playing football) and the mean value for Stage 2 (13 years of playing football) could be used for Stage 2. Continuing this logic, we arrived at 15 years for Stage 3 and 20 years for Stage 4.

Additional data was required to plot the strain-life curve in Fig. 6. To determine the G-levels and number of impacts to the head, data from Mihalik *et al.*^36^ was used to garner the number of head impacts in 1 year. For an offensive lineman, the number of head impacts per year was 10,128 including all practices and games. Clearly, much uncertainty exists when considering different levels of play (high school, college, and professional), different teams, and difference activities in a day, but for this demonstrative example, it is assumed that 10,128 is the number of plays for an offensive lineman that will be used with the Mez *et al.*^35^ and McKee *et al.*^31^,^33^,^34^ data to determine the number of head impacts over 7, 13, 15, and 20 year intervals. Once the number of head impacts over the intervals is associated with the p-tau accumulation levels, the stress or strain amplitude for the strain-life curve must be determined. Determining the stress or strain amplitude is difficult due to an absence of information in the literature; therefore, the G-level data from Funk *et al.*^16^ is used to estimate the amplitude.

As mentioned previously, Funk *et al.*^16^ quantified the G-level impact magnitudes for different positions per aes finding that the offensive linemen experienced the most head impacts. Within the framework of this study, each value was proportioned for the number of impacts per ae by the total number of impacts per ae as reported by Funk *et al.*^16^ and then multiplied that percentage by the number of head impacts per year as reported in Mihalik *et al.*^36^ for each G-level. Essentially, the total number of head impacts (10,128) from Mihalik *et al.*^36^ are binned according to the percentage of particular G-level impacts. With the binned information, the total number of total impacts can be correlated to the G-levels for each tau protein accumulation damage stage level as shown in Fig. 6. Figure 6 further illustrates that offensive linemen experience mostly HCF confirming that HCF is the more dominant mechanism associated with the development of CTE in offensive linemen, who experience more low amplitude, but high frequency impacts; however, another assumption is underpinning this result. When the four tau protein accumulation stage levels were incorporated into the model, the data was based on post-mortem analyses; however, the basic assumption in Fig. 6 is that all of the damage occurred during the years of playing, which is not true, as mechanical creep over time adds to the damage found in the brains of the deceased players.

### Quarterback

**Figure 7:**
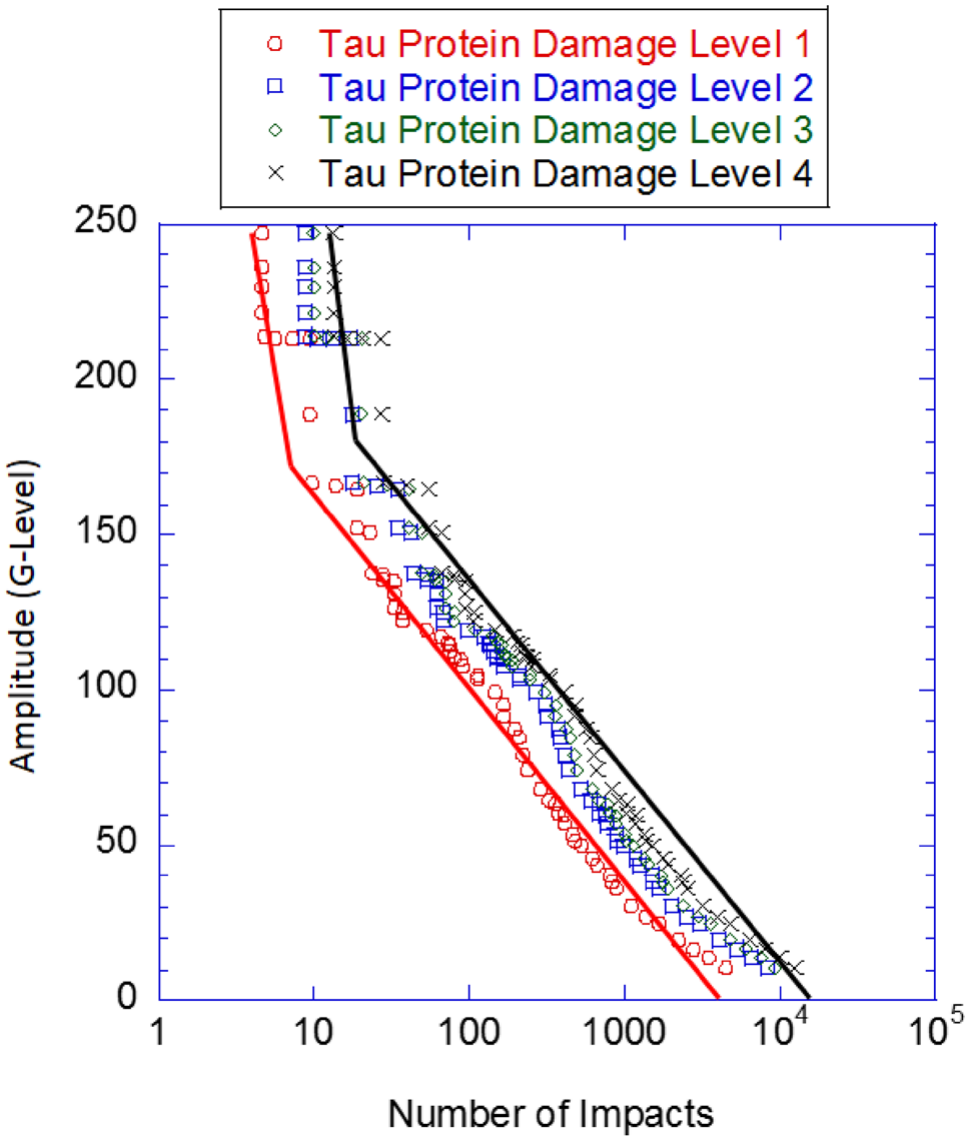
The fatigue-life curve of a quarterback showing the different levels of p-tau accumulation from the different stages defined by McKee *et al.*^31^,^33^,^34^ and Mez *et al*.^35^ Data for the plots were garnered from Crisco *et al*.,^9^,^10^ Broglio *et al*.,^7^ Funk *et al.*,^16^ McKee *et al*.,^31^,^33^,^34^ and Mez *et al.*^35^

As aforementioned, offensive linemen incur HCF regime related damage but a skill position such as a QB incurs LCF regime related damage. Figure 7 shows the strain-life curve for the brain of QBs to illustrate the difference from offensive linemen as shown in Fig. 6. Mihalik *et al.*^36^ did not study the QB position; however, Crisco *et al.*^9^,^10^ and Broglio *et al*.^7^ did study head impacts to QBs, and found that QBs average 307 head impacts per year. Crisco *et al.*^9^,^10^ and Broglio *et al.*^7^ also studied offensive linemen and reported fewer head impacts (798) than Mihalik *et al.*,^36^ who reported 10,128 head impacts; however, the lower measurements from the accelerometers used by Crisco *et al*.^11^,^48^ and Broglio *et al.*^7^ were of a greater amplitude than those reported Mihalik *et al.*^36^ As such, in order to compare Figs. 6 and 7, the ratio of plays from Crisco *et al*.^9^,^10^ and Broglio *et al*.^7^ for QBs to offensive linemen was multiplied by the total number of plays from Mihalik *et al.*^36^ to get 3896 head impacts for the QB.

The fatigue curve (Fig. 7) for the QB position indicates that skill positions incur the most damage in the LCF regime. Further, the transition load level from LCF to HCF occurs at the 170 G-level which concurs with the statement by Crisco *et al*.^10^ that QBs and RBs exhibited the greatest G-level amplitudes during impacts when compared to the other positions. The number of cycles to the LCF/HCF transition for Damage Levels 1 was 9 head impacts at or above 170 G’s, and the number of cycles required to transition from LCF/HCF at Damage Level 4 was 30 impacts at or above 180 G’s. Although no LCF regime was observed in Fig. 6, if an offensive lineman were to experience impacts above the QB LCF/HCF transition levels, then one could anticipate that offensive linemen could experience LCF damage; however, since their impact magnitudes are typically much lower than QBs, offensive linemen mostly experience HCF. Although Funk *et al.*^16^ only published the detailed G-levels for offensive linemen and QBs, trends from their results indicate that along with QBs, the RBs, WRs, TEs, and DBs will also experience both the LCF and HCF regimes; however, along with offensive linemen, the defensive linemen and LBs the majority of p-tau accumulation probably occurs in the HCF regime.

### Damage Growth in NonLiving Materials and Living Brains

**Figure 8:**
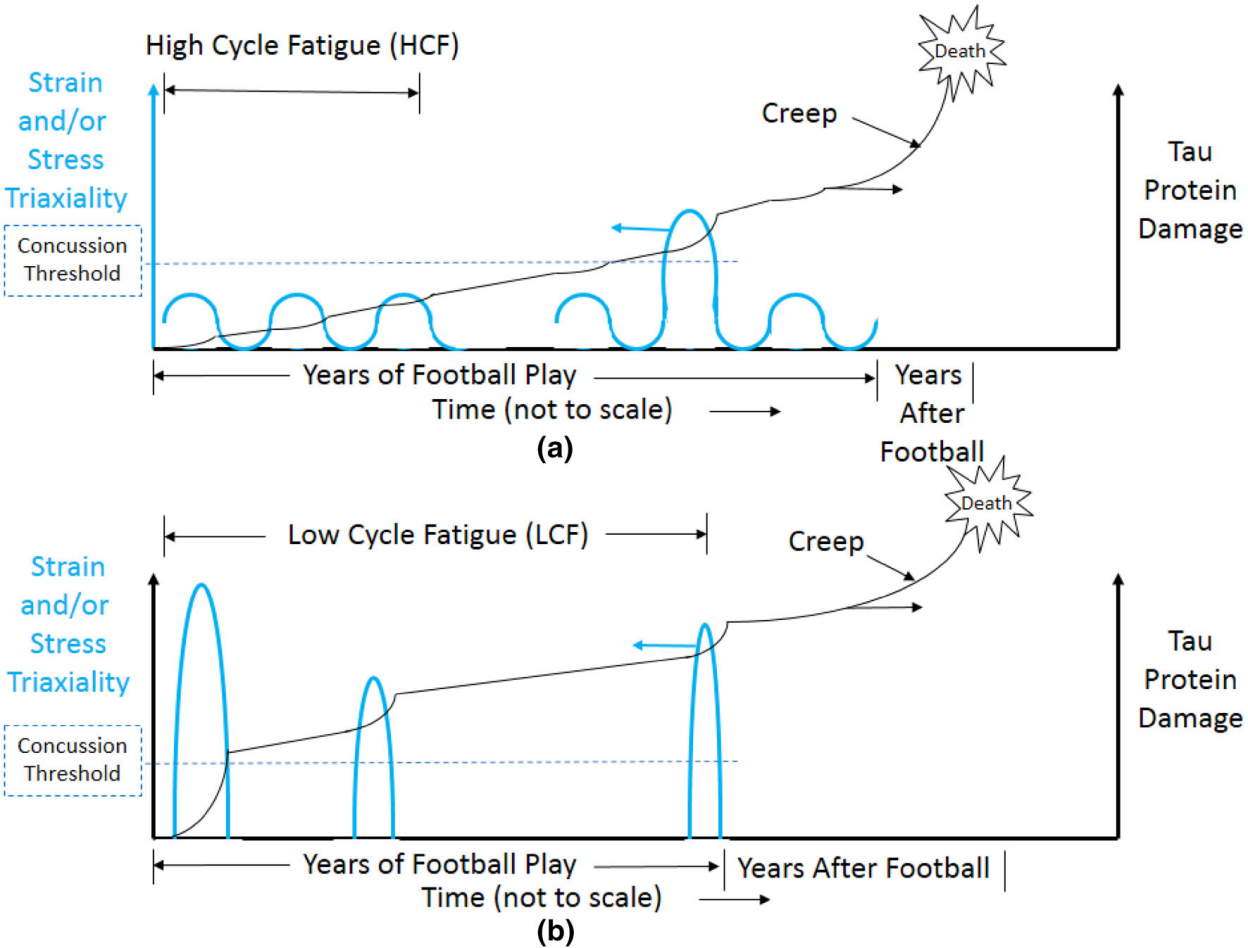
Hypothetical scenarios of two players at two different football positions: (**a**) lineman and (**b**) quarterback. In (**a**) for the lineman, more low level subconcussive impacts occur with most operating in the high cycle fatigue (HCF) regime with an overload just once in a while. Most of the creep damage grows after football until the person dies. In (**b**) for the quarterback, more high level concussive impacts occur with most operating in the low cycle fatigue (LCF) regime. Similar to the lineman, most of the creep damage to the running back grows after football until the person dies.

In Figs. 6 and 7, the damage from the accumulation of p-tau resulted is reported only in terms of fatigue. Realistically, creep associated to p-tau accumulation after playing football also performs a role to cause damage as denoted from McKee *et al.*^31^,^33^,^34^ and shown in Fig. 3. In Fig. 8, the histories of the different players and their respective positions can be somewhat correlated with the levels of damage to the brain reported by Mez *et al.*^35^; however, of note, the study by Mez *et al.*^35^ does not directly identify the brain of each player examined by the position of the player increasing the level of uncertainty in the analysis. Despite this quantitative caveat, the qualitative trends identified in the Boston University studies (e.g. Mez *et al.*^35^) still hold, and as such, some of the assumptions made in the current study, while reasonable, are not fully validated.

The brain consists of at least two networks of recursively branching structures: the blood vessels and the neural processes of axons and dendrites. In addition, the brain contains tubes within tubes: microtubules within the fluid-filled neurites that transport chemicals out and back to synaptic terminals. The mechanical properties at a subcellular scale are very nonuniform and like the neurons themselves are likely to be highly anisotropic and heterogeneous. Therefore, a detailed, multiscale model of brain mechanics will necessarily explore the points of particular vulnerability and respond to experimental data from animal studies that do not yet exist. Despite our present paucity of knowledge there exist intriguing data from other related areas of investigation. For example, Da Mesquita *et al.*^11^ exploit the recent rediscovery of meningeal drainage vessels that operate in parallel with the venous drainage. In transgenic mouse models of Alzheimer’s, disruption of the meningeal drainage system compromises the ability of the venous drainage to remove macromolecules and leads to amyloid- accumulation.^41^ Compromise of the drainage systems may of course lead to changes in CSF density and pressure and a change in the hydrostatic stress state of the brain making the brain more susceptive to creep. Loss of brain volume is also a characteristic of advanced CTE.

Although p-tau accumulation is associated with microtubule damage repair, agglomeration of misfolded p-tau into fibrils is pathological, and its precise effects are not understood. We speculate that these fibrils may disrupt the cytoskeleton and possibly extracellular matrix. One recent study has found that *neurofilament light* can be detected in the blood and spinal CSF of a particular group of Alzheimer’s patients many years before there is behavioral impairment.^11^ The authors suggest that *neurofilament light* is also likely to be associated with brain damage in TBI, and if so, should be investigated in football players and in animal studies.

There are a number of pathologies collectively amyloidosis in which plaques of accumulated proteins accumulate and change the properties of the tissue. The lens of the eye is an interesting model in that the progressive stiffening of the lens with age is due to the agglomeration of proteins that easily stick together to form fibrils. A recent paper has found that a particular steroid molecule, lanosterol, can dissolve these protein plaques and reverse the course of lens stiffening. While it is unknown what the mechanical effects of tau fibrils is in CTE, the studies in the lens are suggestive of work that needs to be undertaken.

At a greater length scale, the axons of projection neurons are organized into a number of nerve tracts that traverse the brain both anteroposteriorly, radially from cortex to subcortical nuclei and back, and laterally between the two cerebral hemispheres. Large, long distance axons may be particularly vulnerable to mechanical insult and it would be interesting to examine how tau concentration is related to nerve tract terminations. All of these, and many other molecular neurobiological issues that we do not have space to discuss here, are suggestive of what a next-generation multiscale model might contain to examine detailed mediating mechanisms in CTE.

Given the unknown multiscale mechanisms, this study introduces a “first order” mechanical damage framework^19^,^20^ that was used to model the deformation mechanisms related to the progression of p-tau protein accumulation and damage found in the brains of professional football players as reported by McKee *et al.*^31^,^33^,^34^ and Mez *et al.*^35^ Different amplitudes and frequencies of impacts arise from different player positions and time after football that give rise to the mechanical loading conditions of fatigue, overloads, and creep. Based on the damage model incorporated in this study, skill positions, such as QB, are more susceptible to damage from LCF loads; whereas linemen are more susceptible to damage from HCF loads. Examples of a QB and a lineman were used to illustrate the corroboration of the damage model proposed in this study and the damage levels analyzed from the Boston University data. Three distinct conclusions are provided:

1. An ISV model with three physically motivated ISV rate equations (nucleation associated with the number density, growth associated with the mean size, and coalescence associated with the *NND*) has been correlated to the damage progression found in the brains of deceased NFL players donated to Boston University. The strong correlation indicates that the different mechanics notions of nucleation, growth, and coalescence are key deformation mechanisms in brain damage progression.
2. Three different mechanical loading conditions (overloads, fatigue, and creep) contributed to the p-tau accumulation and damage nucleation, growth, and coalescence in the brains of the deceased NFL players.
3. Different football player positions were identified with various mechanical loading conditions. Skill position players, like QBs, incurred mainly LCF damage; whereas, linemen incurred mostly HCF damage.
